# Designing and Delivering a Dissemination Programme With Co‐Researchers With Intellectual Disabilities

**DOI:** 10.1111/jar.70313

**Published:** 2026-10-02

**Authors:** Olivia Hewitt, Michael Larkin, Peter E. Langdon

**Affiliations:** ^1^ Intellectual Disabilities Research Institute (IDRIS), University of Birmingham Birmingham UK; ^2^ Oxford Health NHS Foundation Trust, Learning Disability Services Oxford UK; ^3^ Institute for Health and Neurodevelopment, Department of Psychology Aston University Birmingham UK

**Keywords:** co‐creation, co‐design, creative methods, dissemination, inclusive research, intellectual disabilities, template analysis

## Abstract

**Background:**

While dissemination is core to the research cycle, producing accessible materials has received scant attention. Having finished a co‐designed project we wanted to share our findings with a range of audiences.

**Method:**

Using the community engaged research dissemination (CERD) framework helped identify a range of dissemination audiences and appropriate materials. Various dissemination materials were co‐created with facilitators. Focus groups and interviews explored people's experiences of taking part in this process and analysed using Template Analysis.

**Results:**

A dissemination matrix collated various dissemination audiences. We created dissemination materials including a film, zine, infographic and easy‐read document. Through focus groups we found that co‐researchers with intellectual disabilities appreciated being meaningfully involved with dissemination and could make a range of engaging materials, reflecting their experiences of the project.

**Conclusions:**

Guidance to help co‐research teams successfully complete this essential research task is offered. Involving co‐researchers in this stage ensured materials were accessible.

## Lay Summary

- It is important to share our research findings with people in ways they find helpful and accessible.
- We used creative methods so people with intellectual disabilities could make materials to tell people what we found in our research. We made an easy read document, a film, a collage, an infographic, an animation and a zine.
- People with intellectual disabilities and other stakeholders enjoyed making these materials. Involving people with intellectual disabilities in this process made sure other people could access the materials.

## 1 Introduction

Sharing research findings is a crucial stage in research broadly, and specifically within knowledge mobilisation. However, within healthcare research, failures to prioritise dissemination continue and are associated with persistent health inequalities (Baumann et al. 2023). Dissemination refers to the targeted distribution of knowledge to specific audiences using planned communication strategies (National Institute for Health and Care Research 2025) and may include a wide range of audiences including research participants.

The ethical imperative to share healthcare research findings with those involved in research projects (including co‐researchers) as well as other colleagues, collaborators and clinicians is highlighted by international and national organisations. These include the Declaration of Helsinki (World Medical Association 2024) who mandate that researchers engage participants before, during and after a study to ensure they can engage in understanding and disseminating results and by the NHS Health Research Authority who state that participants have a right to know the findings of the research they took part in (HRA 2026). However, this continues to be a neglected phase of the research process and has been raised as a particular issue within the field of psychology (e.g., Bosma and Granger 2022).

In addition to providing dissemination materials, the appropriateness of these materials must be considered. Disseminating results from research projects in accessible and meaningful ways is key to helping improve the engagement of people with intellectual disabilities in research projects led by universities (St. John et al. 2022).

### 1.1 Dissemination and Inclusive Research

While inclusive research methods such as co‐design, co‐production and co‐creation are increasingly popular, it is unclear whether using such methodologies results in better health implementation or leads to improved health outcomes (Grindell et al. 2022). However, the systematic review by Grindell et al. (2022) found that inclusive research methods did lead to partnership working, the sharing of goals, responsibilities and decision making, an increase in creative approaches and making research findings more relevant, engaging, acceptable and accessible.

Within a co‐design model, Langley et al. (2018) describe an approach to sharing, mobilising and activating knowledge, that they term ‘collective making’. This model suggests different forms of knowledge from various stakeholders can be discovered, shared and blended. This resulting knowledge is used to allow stakeholders to learn about the practical applications of knowledge use and collectively create actionable products. Collective making conceptualises impact across individual, group and systems‐level domains. Our evaluation was designed to examine the first two domains, focusing on participants' experiences and the role of collective making in supporting dialogue and shared understanding among stakeholders. Evaluation of wider systems‐level effects was beyond the scope of this study and is identified as an important area for future research.

Regarding inclusive research with people with disabilities, a recent scoping review (Chapman et al. 2025) found only two studies included co‐researchers in the dissemination of research findings, neither of which involved people with intellectual disabilities. Frankena et al. (2019) specifically considered ‘Using results’ as an inclusive research activity within their consensus statement about inclusive research with people with intellectual disabilities. They suggested holding discussions with co‐researchers about the dissemination of results and evaluating this process. They also considered the use of different publication formats and issues of access and ownership of the data.

Parent‐Johnson and Duncan (2024) address the issue of dissemination in inclusive research teams that involve people with intellectual disabilities. They suggest using ‘multiple means of engagement’ to ensure different ways to access content being shared (e.g., easy read documents), ‘multiple means of representation’ of the materials (e.g., use of infographics alongside research presentations) and ‘multiple means of action and expression’ to consider how research findings can be shared with various stakeholder groups (e.g., the wider intellectual disability community).

Our research team (made up of academics, clinicians, people with intellectual disabilities and other stakeholders including advocates, family members and those managing day services for people with intellectual disabilities) has been working together for 5 years. Together we have used Experience Based Co‐Design (EBCD) to co‐design an anxiety intervention based on mental imagery for people with mild to moderate intellectual disabilities called Co‐MAID (Hewitt et al. 2025). Having used co‐design throughout our research project, we felt it was essential to complete our project dissemination phase in line with co‐design principles. However, there is little specific guidance about how best to do this with co‐researchers who have an intellectual disability.

We therefore adapted the community engaged research dissemination (CERD) framework (Cunningham‐Erves et al. 2020) to guide our dissemination activities. While the CERD is not specifically designed for co‐research teams including those with intellectual disabilities, we adapted the CERD to fit the need of our research dissemination. Using a formal framework helps us ensure that our dissemination was planned, conducted and evaluated in a methodical way and allowed us to consider the principles of ethics, feasibility, utility, communication and community input within each stage of the dissemination process.

### 1.2 Aims

Alongside co‐researchers with intellectual disabilities, we aimed to use an adapted version of the CERD framework to:

1. Identify dissemination audiences and determine appropriate dissemination strategies.
2. Create a range of appropriate dissemination materials (in line with Langley's (Langley et al. 2018) ideas around collectively creating actionable products).
3. Evaluate the experiences of being involved with this dissemination process.

## 2 Method

### 2.1 Positionality

The first author (O.H.) is a clinical academic who has led on the development of the Co‐MAID intervention and has worked as a clinical psychologist with people with intellectual disabilities in NHS services for 10 years. The second and third authors provided supervision to the first author throughout the project which was completed as part of OH's PhD.

The second author (M.L.) is an experienced qualitative researcher interested in people's experience of interventions, the use of creative methods and the process of codesign, particularly, in the context of mental health.

The third author (P.E.L.) has worked with people with intellectual disabilities for more than 25 years within the NHS and other settings. He currently works as a responsible clinician within an NHS community learning disabilities team while also undertaking research as part of his employment with the University of Birmingham. He was the first author's primary PhD supervisor.

### 2.2 Design: Adapting the CERD Framework

The research team used an adapted version of the CERD framework (Cunningham‐Erves et al. 2020) to structure their thinking and produce materials to identify dissemination audiences, determine dissemination strategies, design and implement the dissemination programme (including creating a range of appropriate dissemination materials) and evaluate the work.

The original CERD framework consists of Phase 1 in which a partnership is developed and a dissemination team is formed. Given the long‐standing co‐design nature of our research project, these steps were not necessary for our project team. Therefore, we focused on Phase 2 of the CERD (see Figure 1).

**FIGURE 1:**
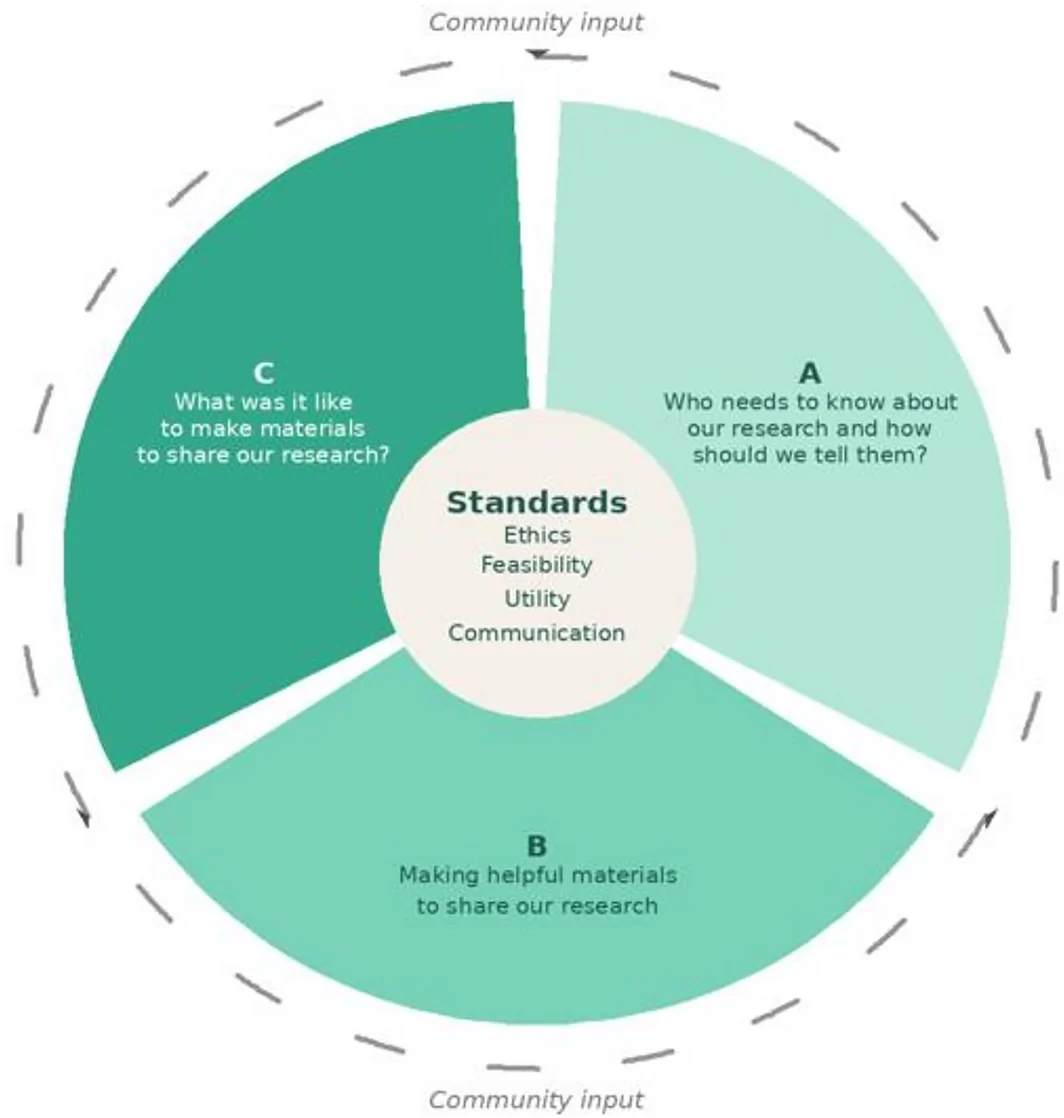
Adapted community engaged research dissemination (CERD) framework (Cunningham‐Erves et al. 2020).

Within Phase 2 (dissemination process) of the original CERD framework, four distinct steps were identified: identify the dissemination purpose, determine dissemination strategies, design a dissemination programme and implement and evaluate this dissemination programme. Within each step, a process occurs iteratively and has been finalised before moving to the next step.

These four steps were consolidated and refined into three steps for our project. This occurred through regular study meetings which involved co‐researchers with intellectual disabilities. The original CERD framework was presented to the study team in diagrammatic form. We spent time thinking about what the words meant and decided to change the words for each step to make them easier to understand. We discussed what parts of the processes were most helpful and relevant for our project.

The original phase A (‘Identify dissemination purpose’) and B (‘Determine dissemination strategies’) were combined as our co‐researchers felt that we already had a clear purpose of the dissemination (which was to share the findings from our research project). However, on discussion we realised that there was some useful thinking that we needed to do around exactly who might want to hear about the results and that different dissemination audiences might want or need different types of materials. For example, the funder or an academic might want a different level of detail and style of presentation of information than someone with a learning disability or a carer who had taken part in the study. Therefore, we consolidated these two original steps into our step A (‘Who needs to know about our research and how should we tell them?’).

The original step C (‘Design dissemination program’) was changed to step B (‘Making helpful materials to share our research’). Our co‐researchers felt that this encapsulated both thinking about and deciding what materials would be helpful and the active role the research team had in physically making these materials alongside support from community partners.

Step D (‘Implement dissemination program and evaluation’) became step C (‘What was it like to make materials to share our research?’). This refined the question and made it specific to our project. While it would have been helpful to evaluate the dissemination programme and understand what wider impact the materials had across different audiences, this was far beyond the scope of our project. However, the research team agreed it was important to evaluate this dissemination phase of the project. They suggested that gathering information about how different members of the research team experienced the process of making dissemination materials using creative methods would be helpful for other research teams to learn about. We therefore designed an evaluation phase to capture this information and decided to share this with other researchers through this journal article.

We agreed that the five standards (utility, feasibility, ethics, communication and community input) that underpinned Phase 2 had been important in our project up to this point and should be retained.

Examples of how these standards were incorporated throughout the three stages of the project are given in Table 1.

**TABLE 1:** Details of Focus Groups and Individual Interviews.

Method of interview	How conducted	Participants	Date conducted (weeks after research day)
Focus Group 1	Face to face	3 people with intellectual disabilities	2 weeks
Focus Group 2	Face to face	5 people with intellectual disabilities	2 weeks
Focus Group 3	Face to face	2 people with intellectual disabilities	2 weeks
Focus Group 4	Online	2 stakeholders and 2 family members	3 weeks
Individual interview 1	Online	1 stakeholder	3 weeks
Individual interview 2	Online	1 facilitator	4 weeks
Individual interview 3	Online	1 facilitator	4 weeks

### 2.3 Sharing Our Research Day

A ‘Sharing Our Research’ Day was planned by the research group with substantial input from co‐researchers with intellectual disabilities. The planning meeting decided on the location of the day to ensure a familiar and accessible venue. Issues around catering and room locations were considered. We decided that having a couple of materials that had already been created and that could be refined during the day would be helpful, alongside several sessions to create materials from scratch. In addition, we wanted to capture the process of the day and so we invited a professional illustrator to produce a live animation. A list of people we would like to invite to the event was compiled to include people who had been involved in previous phases of the project as well as new people who could provide a fresh perspective on our work. We decided to use strategies that had been helpful at previous workshops such as allocating people to mixed groups (people with intellectual disabilities, stakeholders, family members and support workers) and giving each group a colour and Coloured Name Badge.

Four separate activities in different rooms were planned:

- Creating a storyboard
- Making a zine
- Refining existing materials (an infographic and easy read document)
- Making a short film about the project

The Infographic and easy read document are presented in Supplementary Materials 1 and 2. The Storyboard activity was too complicated for participants to engage with and instead, people made a collage of a positive mental image. Participants were allocated to one of four groups and each group took part in every activity. Activities lasted for 45 min each. We had a long lunch break (food provided) halfway through the day. We had a celebration cake to say thank you to everyone for their hard work on the project.

## 3 Procedure

### 3.1 Aim 1—Identify Dissemination Audiences

This dissemination phase of our research project involved a research team made up of academic researchers, clinical academics, co‐researchers with mild to moderate intellectual disabilities and a group of stakeholders (consisting of service managers, advocates and family members of a person with intellectual disability). Co‐researchers with intellectual disabilities had been actively involved throughout several earlier stages of the research project including applying for funding, conducting various research projects, co‐designing an intervention and were now keen to be involved in the dissemination stage to share results of their previous work. The research team had worked together for several years and were comfortable in challenging each other in meetings and asking for clarification.

A range of adaptations were made to ensure accessibility for all researchers, including using accessible and easy‐read written information, presenting information in multiple formats (e.g., visual and audio), giving additional time for members to respond, prompting for areas of disagreement, paying attention to nonverbal cues from members and using such occasions to enquire further about confusion or disagreement. We also made use of printed materials which members could point to or raise to indicate that they were happy (either an A4 size green ‘thumbs up’ symbol or a tick mark), unsure (a large yellow question mark) or unhappy (a red ‘thumbs down’ symbol or cross mark). During regular research meetings, dissemination of findings was a regular agenda item, allowing us to think from the start about the importance of dissemination and how this could be done most helpfully for the project.

During our research meetings, we used the CERD framework to guide our thinking about dissemination. In these meetings, we compiled and refined the dissemination matrix, discussed what dissemination materials had been produced for the various audiences since the last meeting and how best these could be shared and agreed what results should be presented (and by whom) at upcoming conferences. Co‐researchers and stakeholders did not wish to present results in person at conferences, so we discussed alternative ways of ensuring their voices were heard directly, that is, through presenting verbatim quotes, film clips of them talking about their experiences and showing copies of creative materials such as the zine. We also decided that we needed to make our own materials to share our research and that we required some professional help to allow us to do this, specifically regarding live animation, filming and the creation of zines.

### 3.2 Aim 2—Create a Range of Appropriate Dissemination Materials

Together the research team planned a ‘Sharing Our Research Day’ in which we could come together with support to create a range of dissemination materials. We thought carefully about who to invite and what creative methods we wanted to use to produce these materials, considering ideas from Langley et al. (2018) around collective meaning making. We also liaised with organisations, such as the local college and other community learning disability organisations, to support our ‘Sharing Our Research Day’ and who may benefit from our dissemination materials.

### 3.3 Aim 3—Evaluate the Experiences of Being Involved With This Dissemination Process

Following the ‘Sharing Our Research Day’, a series of four focus groups and three individual interviews were conducted with 17 people who had attended the ‘Sharing Our Research Day’. These people included stakeholders, co‐researchers with intellectual disabilities and facilitators and evaluated their experiences of collaboratively creating dissemination materials. Focus groups and interviews were recorded, transcribed and analysed using Template Analysis. All focus groups and interviews took place over 2 weeks in October 2025, thus ensuring they occurred between 2 and 4 weeks after the ‘Sharing Our Research Day’. This time frame was chosen as it allowed participants some time to reflect on the day and how they had experienced it and discuss this with other people in their network, but soon enough after the event that recall would not be significantly impaired.

A semi‐structured interview schedule was used to guide the discussion and topics included ‘How did we share our research findings with other people?’, ‘What did you like about how we shared our research findings?’, ‘What could we have done better/differently when telling people about our research?’, ‘How did you find making dissemination materials together?’, ‘What dissemination materials did you like or not like, and why?’. An easy read version of the interview schedule itself was not made available to the participants. A range of generic (e.g., Can you tell me more about that? What was bad about that?) and specific prompts (e.g., Why did you prefer the zine to the film? Why did you pick that image to include?) were used to elicit more detailed responses. In addition, physical copies of the different dissemination materials (e.g., easy read document, infographic, zine etc.) were present during the interviews and focus groups to participants could refer back to them and give examples to illustrate their opinions about the materials.

Interviews were analysed by a single researcher (O.H.) using Template Analysis (Brooks et al. 2015). A range of strategies were employed to enhance credibility including close documentation of all eight versions of the template (alongside documenting any changes and why they were made which were discussed in supervision), keeping a reflexive log throughout the process and presenting results back to some participants to ensure results resonated with their experiences. All themes were grounded in multiple quotes across a range of participants. While initial coding was conducted by a single researcher, results were discussed within the whole research team (including co‐researchers with intellectual disabilities) as well as in individual supervision allowing for these to be explored and tested over multiple iterations. The template was based on aspects of Frankena et al.'s (2019) consensus statement (which sets out a number of attributes of good inclusive health research), specifically focused on two areas, ‘The Ethos of Inclusive Research’ and ‘Using Results’Frankena et al. (2019, 5) describe the ‘Ethos of Inclusive Research’ as ‘a certain mindset’, which is characterised by (among other things): discussing, understanding and respecting cultural, representational differences, personal biases and power relationships; transparency of decision making and ensuring information is accessible to all. The attribute of ‘Using Results’ includes discussing with team members how results will be disseminated in an accessible manner; reporting on the process and added value of inclusive health research and evaluating the dissemination of results.

Additional template items arose from the transcripts. Template Analysis was chosen as it is a pragmatic and flexible tool allowing particular attention to be paid to material corresponding to the existing literature (as captured through Frankena et al.'s (2019) consensus statement), while allowing flexibility for novel and unexpected areas to be identified.

Ethical approval for interviewing co‐researchers and stakeholders about their experiences of disseminating research was given by the West of Scotland Research Ethics Committee (ref: 24/WS/0022). All participants in focus groups and interviews gave written, informed consent to take part and for the use of anonymised quotations to be used in this publication. To ensure informed consent was obtained to take part in this research, accessible information sheets and consent forms were provided. These used plain English and were enhanced with symbols and pictures in an easy‐read format. Information about the study was provided in chunks and understanding was checked at each stage before going on to the next part of the information sheet. Researchers assessed the participants' capacity to take part in the research in line with the Legislation.gov.uk (2005), assessing their ability to understand and retain information pertinent to the decision to take part in the research, the ability to weigh up this information as part of the decision‐making process and to communicate this decision to the researcher. Consent was assessed throughout the research process, with participants being reminded of their right to withdraw and researchers being mindful of verbal and non‐verbal indicators that a participant may be reluctant to engage with any aspect of the research and checking consent at such points.

## 4 Participants

### 4.1 Aim 1—Identify Dissemination Audiences

Co‐researchers with intellectual disabilities and stakeholders were part of the ongoing research team. This generally consisted of a core group of six people with mild to moderate intellectual disabilities, aged 20s–60s, who were all White British and who had a range of additional mental and physical health disorders. The stakeholder group consisted of a core group of five people, all of whom were White British, aged 40s–70s and whose role included family member of a person with intellectual disability, advocate and service manager for intellectual disability services.

### 4.2 Aim 2—Create a Range of Appropriate Dissemination Materials

The ‘Sharing Our Research Day’ was attended by 28 participants (7 facilitators, 7 stakeholders [including two support workers] and 14 people with intellectual disability). Twenty participants were female, 26 were White British, one was Asian and one was mixed White and Asian ethnicity.

### 4.3 Aim 3—Evaluate the Experiences of Being Involved With This Dissemination Process

Seventeen participants took part in focus groups and interviews to share their experiences of taking part in the dissemination process (see Table 1). Both focus groups and individual interviews were pragmatically used to collect data from as many participants as possible within a short time after the ‘Sharing Our Research Day’ event. Therefore, the same semi‐structured interview schedules were used for all data collection. Data saturation was not formally evaluated and the sample size was determined pragmatically.

Participants in Focus Groups 1, 2 and 3 all had a mild intellectual disability, all were of White ethnicity, seven were women and their ages ranged from the 20s to 50s. They had a range of additional physical and mental health conditions including anxiety disorders, cerebral palsy, hearing impairment and epilepsy. People with a range of additional comorbidities were included throughout this research project to reflect the additional complexities of people with intellectual disabilities likely to use the Co‐MAID intervention within NHS settings.

Participants in the Focus Group 4 and the three individual interviews had a range of roles within intellectual disability services including assistant psychologist, advocate, day service manager and family member of a person with intellectual disability. Their ages ranged from the 20s–70s, all were of White ethnicity, six were women and they had additional physical and mental health conditions including anxiety disorders. The two family members in Focus Group 4 had not been involved in previous phases of the research project.

Individual interviews and focus groups were conducted by the same member of the research team. This led to some complexities regarding power dynamics within the interviews. To address these and the subsequent acquiescence and social desirability that may be elicited, the following strategies were employed; explicitly naming the pre‐existing relationship and being transparent about the power imbalance this could lead to and asking the participant if there is anything that could help reduce this (as well as giving some suggestions). Other strategies included conducting interviews at the person's home or their preferred setting (e.g., day centre), explicitly giving participants permission to disagree and probing for difficult or negative experiences to be described, using open ended questions and prompts, allowing for silence and paying attention to pacing to allow more ambiguous or difficult responses to be formulated and communicated and attending to non‐verbal cues such as discomfort or confusion and using these cues to offer a break or check consent. Additionally, the use of co‐researchers in developing the semi‐structured interview schedule and in analysing results is in line with good practice in inclusive research (Johnson and Walmsley 2003). Separately, the results of the analysis (once compiled and anonymised) were presented back to the research team (several of whom had taken part in the interviews and focus groups). This constituted a process of member checking, enabling participants to challenge or correct the researcher's interpretations. The researchers used various tools to facilitate reflexivity including journaling and supervision to understand how their prior relationship with participants and their role within the research project may shape their interpretation of the results.

## 5 Results

### 5.1 Aim 1—Identify Dissemination Audiences

Through discussions in the research team, a dissemination matrix table was produced (see Table 2). A simplified version with colour coding for different audiences, including photos (e.g., of published articles) and symbols was presented at research meetings to ensure accessibility for co‐researchers with intellectual disabilities. This document was revisited and updated throughout the project and continues to be added to as we implement aspects of our dissemination strategy as a research team.

**TABLE 2:** Dissemination matrix for the Co‐MAID project.

Dissemination audience	Aim of dissemination strategy	How to implement dissemination strategy	Date implemented/tangible outputs
Research funders	Communicate to funders that all research outputs identified in project plan have been achieved	Annual funding report delivered to funder detailing community engagement	Funding report delivered March 2023, March 2024, March 2025
	Ensure funders are aware of range of research outputs	Detail research outputs (academic publications, community engagement events etc.)	Funding report delivered March 2023, March 2024, March 2025
	Funders are aware current project has been successfully delivered	Submit final contractual output	Report delivered to funder January 2026
	Provide sufficient evidence for initial promise of intervention to secure additional funding for subsequent feasibility study	Funding application for feasibility study submitted October 2024	Funding for feasibility study secured August 2025
Commissioners of NHS clinical services	Communicate potential of new intervention	Present findings through meetings/channels attended by commissioners such as local Integrated Care Board	
Research host organisation/project sponsor	Aware of status of research participants and successful recruitment to target	Update Edge Research Management portal	Recruitment closed on Edge 1 May 2025
	Ensure accurate records regarding status of clinical trial	Update trials registry ISRCTN 70980810	To update with publication once accepted
	Alert to any ongoing issues with implementing intervention in clinical settings	Regular meetings to feedback with head of Research and Development	Monthly meetings held
Local NHS services (research participants recruited from here)	Ensure services are aware of the intervention that has been delivered to patients	Copy of feedback (easy read) for participants sent to service clinical lead and uploaded to electronic patient notes	12 August 2025
	Ensure relevant electronic patient notes updated in line with department/trust standard operating procedures	Update NHS clinical notes for each participant and compile and upload closing letter summarising individual progress through intervention sessions.	By 30 June 2025
	Ensure relevant electronic patient notes updated in line with department/trust standard operating procedures	Update risk information as appropriate on patient electronic notes	By 30 June 2025
	Ensure relevant electronic patient notes updated in line with department/trust standard operating procedures	Ensure onward referrals made at end of intervention or appropriate closing document completed and patients discharged from service.	By 30 June 2025
	Ensure services are aware of any issues in the delivery of the intervention (implications for other research studies recruiting in the department)	Regular presentations (every 3 months) to update department on study at department research meetings	Ongoing
	Ensure services are aware of the intervention that has been delivered to patients	Clinical supervision with service lead to discuss intervention and any issues with participants (throughout duration of intervention delivery)	Completed by 30 June 2025
Community organisations for people with intellectual disabilities (e.g., learning disability partnership board, self‐advocacy groups)	Alert organisations to co‐designed aspect of intervention plus initial clinical results	Send electronic and hard copies of creative dissemination materials Offer to deliver an information session to community organisations	Ongoing
	Alert organisations to co‐designed aspect of intervention plus initial clinical results	Attend local meetings and deliver presentation to describe the project (to be delivered by OH, stakeholders and co‐researchers with intellectual disabilities). Include creative dissemination materials as part of presentation	Ongoing
	Alert organisations to future research opportunities around Co‐MAID	Provide information about upcoming feasibility study	Ongoing
Local/national charities	Alert organisations to co‐designed aspect of intervention plus initial clinical results	Send electronic and hard copies of creative dissemination materials offering to deliver an information session to community organisations	
	Alert organisations to co‐designed aspect of intervention plus initial clinical results	Attend local meetings and deliver presentation to describe the project (to be delivered by OH, stakeholders and co‐researchers with intellectual disabilities). Include creative dissemination materials as part of presentation	
	Alert organisations to future research opportunities around Co‐MAID	Provide information about upcoming feasibility study	
Stakeholders involved as research participants	Feedback aggregated results of study to research participants in accessible format	Create summary sheet of each project and send to all participants who requested feedback	Summary sheet
	Feedback aggregated results of study to research participants in accessible format	Hold community event to disseminate research findings	Event held at community venue 26 March 2025 attended by 12 participants
	Feedback aggregated results of study to research participants in accessible format	Hold community event to disseminate research findings	Event held at community venue 6 September 2022 attended by 20 participants
People with intellectual disability involved as research participants	Feedback aggregated results of study to research participants in accessible format	Create easy read summary sheet of each project and send to all participants who requested feedback	Easy read summary sheet
	Feedback aggregated results of study to research participants in accessible format	Hold community event to disseminate research findings	Event held at community venue 26 March 2025 attended by 12 participants
	Feedback aggregated results of study to research participants in accessible format	Hold community event to disseminate research findings	Event held at community venue 6 September 2022 attended by 20 participants
Clinicians working in field of intellectual disability	Disseminate initial findings including implications for clinical practice (i.e., potential for use of mental imagery components)	Present findings regarding intervention across formats regularly accessed by clinicians	Present information at regional Special Interest Group meeting (5 July 2023) (14 May 2025) Provide training to University of Oxford Trainee clinical psychologists 3‐h session in person 6 January 2026 Provide training to University of Southampton Trainee clinical psychologists 3‐h session in person 17 April 2026 Provide training to Birmingham CLDT 3‐h session online 22 January 2026
	Share best practice/innovation (co‐design of psychological intervention)	Publish academic article describing how to adapt Experience based Co‐Design to develop intervention	Hewitt et al. (2025)
Academics working in field of intellectual disability	Update on progress of Co‐MAID study	Present at national conference	20th Seattle Club Conference
	Update on progress of Co‐MAID study	Present at national conference	21st Seattle Club Conference
	Update on progress of Co‐MAID study	Present at international conference	International Association for the Scientific Study of Intellectual and Developmental Disabilities 17th World Congress
	Synthesis literature around inclusive health research for this population	Publish academic article describing systematic review	Hewitt, Langdon, Tapp, et al. (2023)
	Share evidence base (describe phenomenology of mental imagery in this population)	Publish academic article to report the phenomenology of mental imagery in people with ID	Hewitt, Langdon, Hales, et al. (2023)
	Share best practice/innovation (co‐design of psychological intervention)	Publish academic article describing how to adapt Experience based Co‐Design to develop intervention	Hewitt et al. (2026)
	Share best practice/innovation (disseminating research findings with co‐researchers)	Present at national conference	22nd Seattle Club Conference
	Share novel intervention as addition to literature	Publish academic article describing Single case Experimental Design and process evaluation of intervention	Hewitt et al. (2026)
	Share best practice/innovation (disseminating research using creative methods)	Publish academic article describing how to use creative methods to produce accessible dissemination material and how this process was experienced by co‐researchers	Current manuscript
Academics working in field of mental imagery	Share evidence base (establish ability of people with intellectual disability to engage with all aspects of mental imagery)	Publish academic article describing systematic review	Hewitt, Langdon, Tapp, et al. (2023)
	Share evidence base (describe phenomenology of mental imagery in this population)	Publish academic article to report the phenomenology of mental imagery in people with ID	Hewitt, Langdon, Hales, et al. (2023)
	Update on progress of Co‐MAID study	Present at national conference	52nd Annual Conference of British Association for Behavioural and Cognitive Psychotherapies
	Update on progress of Co‐MAID study	Present at national conference	53rd Annual Conference of British Association for Behavioural and Cognitive Psychotherapies
	Describe initial evidence for use of mental imagery within clinical settings for people with intellectual disabilities and potential impact on clinical symptoms of anxiety	Publish academic article to report initial findings of using Co‐MAID in NHS settings	Hewitt et al. (2026)
	Develop specific measure of mental imagery in people with intellectual disabilities (mental Imagery Questionnaire for people with Intellectual Disabilities; MIQ‐ID) and test validity of this	Publish academic article to report development and testing of MIQ‐ID	Hewitt et al. (in preparation)

The dissemination matrix highlights the breadth of audiences that may need to be considered within inclusive research projects. While dissemination activities aimed at project stakeholders, research participants, funders and host organisations were relatively straightforward to implement because existing communication pathways were already established, dissemination to wider audiences such as commissioners, charities and community organisations required additional planning and remained ongoing at the time of writing. The matrix therefore functioned as a dynamic planning tool, helping the research team identify gaps in dissemination activity and prioritise future work. Research teams using a similar approach prospectively should expect the matrix to evolve throughout a project as new dissemination opportunities emerge and priorities change.

The dissemination matrix (a) identifies dissemination audiences and (b) determines appropriate dissemination strategies for these audiences. It details the wide range of different dissemination audiences who may need to hear about the results of an inclusive research project. For each audience, it considers what the aim of the dissemination strategy is, how the specific dissemination strategy can best be implemented and for the Co‐MAID project, detailed the date on which this strategy was implemented, along with details of any tangible outputs produced.

### 5.2 Aim 2—Create a Range of Appropriate Dissemination Materials

Table 3 details the dissemination programme that was created by the whole research team. This includes the six different creative materials that were produced by the research team and an external illustrator. It also details how these materials have already been shared and plans for making these available through a website which is currently being produced.

**TABLE 3:** A description of the creative materials produced by the research team.

Dissemination material	Brief description of material	Who made this?	How will it be shared?
Infographic	Single page document that visually captures key points of research project, using colour, images and short phrases/single words.	Professional illustrator	Presented at conference To be shared on website
Film	Several short (1–3 min) clips of people with intellectual disabilities and stakeholders talking about their experiences of having anxiety, strategies to manage this and their experiences of being involved in the Co‐MAID research project as co‐researchers.	Local college students, Stakeholders and co‐researchers with intellectual disabilities	Presented at conference To be shared on website
Zine	Self‐published A4 size booklet containing text, words, drawings and collage to capture experiences of co‐research in the project	External facilitator, stakeholders and co‐researchers with intellectual disabilities	Hard copies shared with attendees of ‘Sharing Our Research Day’ Presented at conference To be shared on website
Collage	A series of individual A4 sized documents incorporating materials such as paper, pictures, text, drawings and stickers to reflect positive mental images created by co‐researchers	Stakeholders and co‐researchers with intellectual disabilities	Presented at conference To be shared on website
Easy read document	4 pages of A4 containing short, jargon‐free sentences, simple words and clear images (photos and symbols) to summarise the key points of the research project	Stakeholders and co‐researchers with intellectual disabilities	Presented at conference To be shared on website
Live animation	Drawings made in real time during the ‘Sharing Our Research Day’ which capture the key points, conversations, emotion and feel of the event	Professional illustrator	Presented at conference To be shared on website

### 5.3 Aim 3—Evaluate the Experiences of Being Involved With This Dissemination Process

Template Analysis of the semi‐structured interviews identified a total of 14 subthemes which were synthesised into four overarching themes (see Table 4). Pseudonyms have been used in the quotes reported below to preserve anonymity.

**TABLE 4:** Superordinate and subordinate themes from interviews and focus groups.

Superordinate theme	Subordinate theme
Working collaboratively	Networking and working with others
	Sharing our experiences in a supportive environment
	People with intellectual disabilities were meaningfully engaged with the process
Challenges to the process	Not enough time to make materials
	More preparation/structure/direction would have been helpful
	Sensory barriers to engagement
	Being flexible and adaptive when things do not go to plan
Creating a range of different materials was important	We really liked some of our materials—and disliked others
	Creating different materials required different skills
	Different materials have different audiences
The materials we created had personal resonance	Our materials have personal meaning
	We are proud of our contributions
	Being meaningfully involved in creating materials
	We recognise ourselves depicted in the materials

### 5.4 Theme 1: Working Collaboratively

Participants described their sense of working in partnership with other people attending the day and those facilitating activities.

The subtheme ‘Networking and working with others**’** described reconnecting with familiar people and working alongside those who were new to the project:

FG1 PPT3: ‘[We] were put into groups in different colours and we all got get moved around and learned about and spoke with different people’.

Having a mix of familiar people who had worked together previously helped participants to feel comfortable and engage with tasks:

FG4 PPT2: ‘It was a nice mix of people, wasn't it? … I think that helped some of the familiarity that they, they've got faces that they all knew as well’.

PPT4: ‘It makes it a safe space, yeah’.

Having people from a range of different backgrounds working together helped to provide a diversity of opinions and introduce new ideas:

FG3 PPT2: ‘It was good to have it all mixed up together…Because we need new people as well. Yeah, to get to know people a bit more’.

The second subtheme ‘Sharing our experiences in a supportive environment’, described some of the contextual factors required for genuine inclusive research to take place, including feeling psychologically safe to share personal experiences and be vulnerable with others. Participants emphasised the importance of having a collaborative environment in which people worked together or alongside each other to make materials:

II PPT2: ‘Everybody seemed really supportive and friendly with each other and there was some really nice interactions with some participants suggesting to other participants of what they could use for their picture. and lots of nice comments … about how nice their picture looked or how good that looked, or what good idea that was’.

Participants reflected upon the power dynamic between them and facilitators. Some described feeling assured that help was available if required:

FG3 PPT3: ‘No, no, they [facilitators] was they were so kind and so even nice. …. They were helpful’.

However, some participants felt that facilitators or those helping to capture the day, could have been more engaged with the process of co‐creating materials and seemed too detached:

FG4 PPT1: ‘I think I think perhaps [facilitators] needed to be a bit more hands on as well and a bit more helpful in a way…sometimes I felt they could have actually explained a bit more and participated a bit more with our guys … I thought they were a bit hands off’.

Overall, there was a clear sense from participants that ‘People with intellectual disabilities were meaningfully engaged with the process’ of creating dissemination materials:

FG4 PPT2: ‘I think that some of the learning disability young people that I worked with really seem to have grasped what you were trying trying to to get with … So that was really, really good that they … Understood what they what they were, what they were trying to do’.

II PPT1: ‘Yeah. I mean, I think that's [outputs] lovely. I think that's great. You've certainly got plenty there and and and everybody engaged in that really well’.

II PPT2: ‘They just felt like a lot of enthusiasm as well for the tasks, which is lovely. It felt like people wanted to talk about their experiences of taking part … you could feel the energy of people when you're engaging in these creative tasks’.

In summary, Theme 1 demonstrated that participants valued this collaborative work not just as a means of producing dissemination materials, but of developing relationships, confidence and shared understandings. They described learning from each other, contributing in a supportive environment and drawing on different forms of expertise. Meaningful engagement was facilitated when their experiences were listened to, valued and incorporated into the work. This emphasises the relational nature of co‐creation, in which connection and learning from each other are as important as outputs produced.

### 5.5 Theme 2: Challenges to the Process

Participants described various challenges to the process of creating dissemination materials.

In the subtheme ‘Not enough time to make materials’, some felt that there was insufficient time to engage fully with each activity:

FG2 PPT1*:* ‘It was fun, but I don't think it was long enough on each each, each each department … it felt like we were rushing.

Some participants felt that extra time was needed as participants wanted to complete the activity, as well as spending time engaging with others:

FG2 PPT5: ‘That because we weren't all just focusing on what we were doing, we were also talking with one another. … it wasn't just that you were doing an activity’.

In line with other research (Hewitt, Langdon, Tapp, et al. 2023), there was a sense that a lack of time and other resources could be a barrier to genuine inclusive research in this project.

Some facilitators and participants felt that ‘More preparation/structure/direction would have been helpful’ ahead of the day would have helped ensure all felt confident in knowing what they were doing and who was attending the day:

II PPT2: ‘It might have been helpful for… all the facilitators to have met beforehand to work out exactly how it was going to flow … I wasn't sure exactly who was the facilitators at one point and who were the participants so. I was a little bit of trying to figure out that at the start, which was fine, but maybe could have been a bit smoother’.

#### 5.5.1 Sensory Barriers to Engagement

The day was held across three rooms and several participants found the rooms to be hot and crowded at times, which could have hindered their ability to engage with activities:

FG3 PPT 1: ‘I got confused which group I was or wasn't…The blue [group] was too many people… I found it too crowded’.

One participant who experienced anxiety found the rooms to be too small and noisy, especially in the break at lunchtime:

II PPT1: ‘It started a little bit wrong when we went for lunch because we're all in quite a smallish room and just it got very noisy, I'm afraid, with some of the other guys all being a little bit silly and being a bit loud’.

#### 5.5.2 Being Flexible and Adaptive When Things Do Not Go to Plan

On the day there were a number of logistic challenges such as some participants arriving late. One of the activities that was planned (a storyboard) proved too complicated for participants to engage with and so facilitators changed this activity to one in which participants used various materials to create depictions of a positive mental image. Facilitators found the flexibility to change tasks to suit participants was helpful:

II PPT3: ‘I just think they're [participants] amazing. I also I think because the day was very much like we went with what worked and it was a bit trial and error and things kind of happened the way they did … it's not like like so you know structured’.

There was an acknowledgement that not all tasks suited all participants, which made having a range of different activities across the day important:

FG4 PPT4: ‘The flexibility for that was really good as well…Jason certainly didn't want to be involved in any of the creative stuff. But when we were talking about like the easy read stuff, …and also with the film, he was he was better able to engage then. So I just guess he doesn't like the creative stuff?’

Meaningful engagement was influenced by practical, organisational and environmental factors. Participants' ability to fully engage with tasks was impaired by limited time, uncertainty around tasks and sensory barriers. The need for facilitators to adapt and be flexible when planned activities were not possible illustrates how genuine co‐creation cannot be achieved through rigid preplanning. Rather, it requires ongoing responsiveness to participants' needs and preferences throughout the research process. This theme illustrates how adequate resources, flexibility and preparation are key to enabling meaningful inclusion.

### 5.6 Theme 3: Creating a Range of Different Materials Was Important

Participants spoke frequently about the importance of having a range of materials that were created and refined during the day as well as in preparing for the day. Having this range of materials was important for a variety of reasons, allowing participants to engage with processes they enjoyed, using particular skills and meeting the needs of various audiences. Having two materials prepared in advance of the day (infographic and easy read document) allowed us to spend time refining the easy read document and considering which audiences might benefit from these materials. It also allowed facilitators to be flexible in their approach on the day, knowing that two dissemination materials had already been produced.

The subtheme We really liked some of our materials—and disliked others captured a range of responses to the different materials we made together.

In one focus group, participants had different experiences of taking part in the films and consequently different opinions on how useful these films might be for others learning about the research:

FG2 OH: ‘And if there was someone who just wanted to find out a bit about our project, what would you recommend they start by looking at out of all these things?’.

PPT3: ‘The filming, I think’.

PPT4: ‘The filming, yeah, yeah’.

PPT3: ‘It's got more information’.

PPT5: ‘Uh, I wouldn't really say that because like I found like with my group, umm, we felt very uncomfortable and we didn't really feel like ourselves. So I felt like it was very hard for us to give the information. So I wouldn't say the format has a lot of information’.

The zine was viewed favourably by many participants. One focus group commented how engaging it was as a material:

FG1 PPT1: ‘I like how it just like, captures different people's unique personalities. It's everyone does it in their own unique way’.

PPT3: ‘It's different … at the same time it looked inviting. Yeah, I think you're right. It's it's engaging, isn't it? Cause it's different and it's colourful and it's bright’.

Another focus group commented on the shared ownership of the zine:

FG4 PPT1: ‘I think she [facilitator] put it together beautifully’.

PPT4: ‘We've produced something physical, haven't we? But it's also got all that emotion in it and everybody's had an equal chance … Everybody is part of that. … And being able to share it. But I think it's having ownership of that. Everybody has equal ownership of that’.

PPT2: ‘I love the zine’.

Participants noted that ‘Creating different materials required different skills’:

FG1 PPT3*:* ‘Some people are crafty, crafty, kind of her people. And like, sticking things and stuff. Whereas other. Yeah, whereas people like me, for instance, aren't as keen and we're better. I prefer writing things and drawing’.

They also acknowledged that ‘Different materials have different audiences’ and that some materials could be used best in combination to illustrate different aspects of the project. Some participants described how an easy read document could be complemented by the zine, providing different types of information about the project:

FG4 PPT4: ‘You need you kind of need them all [different materials]… the easy read document, that's going to be a great one for anyone and everyone to be able to see … then the rest kind of add on to that, they enhance it … the Zine would be the more, about how it felt…’.

In this Theme, participants acknowledged that the process was not about producing a single output but communicating with multiple, diverse audiences. Their discussion about preferred materials, the different skills needed to make them and how suitable these were for different audiences showed an awareness of various communication needs. Creating multiple outputs also allowed for individuals with different strengths to continue while improving the accessibility and engagement of various materials with a range of audiences.

### 5.7 Theme 4: The Materials We Created Had Personal Resonance

Participants described how ‘Our materials have personal meaning’, with the process of creating them being one requiring emotional investment:

FG1 PPT2: ‘They're very beautiful. They're quite personal as well’.

FG 2 PPT4: ‘I found it a bit emotional though. Did you find it emotional doing it? It did make me’.

In the subtheme ‘We are proud of our contributions’, several participants described a feeling of pride and accomplishment in the materials they had created:

FG1 PPT3: ‘I feel good about it and I think a lot of people feel, oh, I feel that that I feel good about it…’.

FG1 PPT4: ‘And there are a lot of people who love them [dissemination materials] and everything made them comfortable and joyful.

FG3 PPT2: ‘It's all brilliant, actually, all the studies. I enjoy, I do enjoy meeting’.

In the subtheme ‘Being meaningfully involved in creating materials’ participants described the value of having a safe space to be creative and share feelings.

Participants described reviewing what we have done in the project and what needed to be done next:

FG1PPT2: ‘We talked about this research and what we need doing [next]’.

It was important for participants to see how their contributions had fed into the materials and been incorporated into what was disseminated to different audiences.

FG4 PPT2: ‘That's it's just nice to see some of the suggestions that that that our group made have have come in as well. So that that looks good’.

The importance of people with intellectual disabilities being actively involved at this stage of research (as they have been throughout the project) was illustrated by one participant:

II PPT1: ‘I don't think it's been too ambitious. I I I think I think it's been lovely. I think it's been so great to involve the guys in this at the end of the day, this is this is for them and about them and hopefully going to help them so. Seeing them involved to sort of be part of this, I think is absolutely great’.

We recognise ourselves depicted in the materials.

Several participants were pleased and excited to see themselves in the dissemination materials, either in the live animation or as part of the film clips:

FG2 OH*:* ‘Anyone notice? Here's that, that is Zadie’.

PPT1: ‘That's me. Thank you’.

PPT5: ‘That that's. That's me. It. That's Jacob. I think because it's got yeah coz it's got his hearing aid in there’.

This raised some concern that everyone's contribution was acknowledged and included in some way:

FG4 PPT4: ‘I'm just wondering what the guys might think if they did it and then their film didn't get so it got left out so I didn't know if … there might be a way to do something even if you incorporate that, I don't know … So that I know it's going to be hard, but just to try and make sure nobody's left behind’.

This led to a discussion and agreement that everyone attending the day should receive a certificate to acknowledge their contribution to the project. In addition, high‐quality hard copies of the zine were also made and one given to everyone attending the day to help them remember the work that had been done.

This Theme suggested that the value of the materials produced went beyond that of dissemination outputs. Participants described feeling proud of their work, recognising their own contribution and having a sense of ownership over the work they had made. Creating materials allowed participants to see their knowledge, experience and identities reflected in the project. From a collective making perspective, the process of creating shared outputs may therefore have served not only to communicate findings, but also to validate contributors' expertise and make their involvement visible in tangible and meaningful ways.

While the theme ‘People with intellectual disabilities were meaningfully engaged with the process’ relates to participants' involvement in the collaborative workshops and decision‐making processes, ‘Being meaningfully involved in creating materials’ specifically refers to contributors' experiences of shaping and recognising their input within the final dissemination products.

## 6 Discussion

Having completed a co‐designed project to develop and test an anxiety intervention, our research team explored ways of ensuring the dissemination of our research was wide ranging and involved people with intellectual disabilities in the design, creating and sharing of research findings. People with intellectual disabilities made engaging, relevant dissemination materials (including audio, visual and image‐based materials which are accessible to those with no literacy and a range of sensory impairments). We produced a wide range of materials which allowed for different aspects of the research project to be communicated. For example, the easy read document gave lots of factual information, while the zine reflected a sense of collaboration and having every person's contribution valued. Gathering feedback from people making the dissemination materials and facilitating this process allowed us to learn more about how this process was experienced. Generally, people enjoyed being part of this creative process and appreciated seeing their contributions being shared widely.

Parent‐Johnson and Duncan (2024) identify that using multiple means of representation (having more than one material that depicts the same information but in a different format) can be helpful. Within our project we aimed to have several different materials that captured similar information (such as an infographic and easy read documents). We also had some materials that captured a different aspect of the project; for example, the collage material reflected the experiences of generating positive images. The zine reflected a sense of belonging and mattering and of working together that co‐researchers described throughout the project and which fits with ideas of engagement.

Participants recognised themselves in materials (as described in the subtheme ‘The materials we created had personal resonance’). This felt ownership is in line with the importance of representation of (multiple) marginalised and underrepresented groups in research (Rabbitte and Adam 2023) (While being mindful of the heterogeneity of people with intellectual disabilities) and with the need for epistemic justice for people with intellectual disabilities (Catala 2020). Materials capturing the experiential or felt sense of being part of the research project are seen as equally meaningful by the research team and valued as such. However, we are aware that some forms of knowledge, such as lived experiences, can be seen as less valuable, especially when relating to people with intellectual disabilities. As a research team we have worked to acknowledge and challenge this form of epistemic injustice, through ensuring those with limited communication skills were able to contribute in different formats to these dissemination materials and that this contribution was valued. We have continued to value and include qualitative methodologies within our project and have focused on those that explicitly explore the lived experience of people with intellectual disabilities. People with intellectual disabilities frequently lack access to shared interpretive frameworks that allow them to meaningfully understand, make sense of and share their experiences with others, which Fricker (2007) labels ‘hermeneutical injustice’. We have sought out and advocated for people with multiple comorbidities who are at increased risk of exclusion from research, such as those with both an intellectual disability and additional disabilities including physical (e.g., cerebral palsy, deaf) and mental health conditions (e.g., anxiety). By including co‐researchers with intellectual disabilities in the adaptation of the CERD and throughout its implementation, we aim to challenge both hermeneutic injustice and contribute to the fight against a deeper injustice often present in frameworks which determine who counts as a knower (Catala 2020).

Within the project there was concern that the contribution of all participants was acknowledged and valued. Participants were represented to different degrees within some of the dissemination materials (e.g., not everyone chose to be involved with the film and some people were recognisably depicted in drawings in the live animation). However, all participants contributed to the zine and the easy read document. To address this, all participants were given a professionally printed copy of the zine to keep and a certificate of participation. These were presented at an informal ceremony at the day centre, photos of which were displayed in the day centre to mark this contribution.

One learning point arose from the storyboard activity, which was unable to be implemented and replaced on the day with a collage activity. Although the activity had been planned collaboratively with co‐researchers with intellectual disabilities, it was more difficult to engage with than anticipated. On reflection, the task required participants to think sequentially, organise ideas into a narrative structure and anticipate how these might be communicated to others, demands that some participants found cognitively challenging. Despite the activity appearing accessible during planning, it was not accessible in practice. Piloting activities with a small group of co‐researchers in advance, ensuring alternative activities are available and building sufficient flexibility into dissemination events to allow adaptation when needed may be helpful advice for other researchers engaging in similar work. However, we view the change in activities as an example of responsive and genuinely inclusive practice, where activities were adapted to participants' needs in real time.

When considering how dissemination was carried out in a genuinely co‐created manner, we found little guidance. However, adapting the CERD framework gave some guidance for the research team and ensured that we focused simultaneously on overarching principles (ethics, feasibility, utility and communication and community input) while we undertook the specific activities of dissemination. People with intellectual disabilities were involved in all stages of thinking about dissemination, including identifying various audiences, discussing what materials we should and should not make, creating these materials with support from others and feeding back their experiences, which ensured that the process of dissemination was genuinely carried out in partnership with our co‐researchers.

Involving facilitators and those with other specialist skills (such as an illustrator) allowed for expertise to be shared with the research team and resulted in professional looking materials, eliciting pride from the co‐researchers. These aspects of dissemination, in line with what we know of inclusive research (Hewitt et al. 2025), are costly in terms of time and money and have resulted in a variety of accessible, engaging and meaningful dissemination materials that reflect the experiences of co‐researchers throughout this project.

Having prepared two materials ahead of the ‘Sharing Our Research Day’ and knowing that these were available for dissemination, meant we felt more relaxed during the day and facilitators felt less worried when activities did not go to plan and needed to be altered. Having a loose framework for the activities which allowed for materials to be taken in different and unexpected ways was a strength of the project. However, ensuring that all facilitators felt as well prepared and confident in their knowledge of all aspects of the research project, as they did in their skills in working with people with intellectual disabilities, would have aided the day.

Despite encouragement from the first author, none of the co‐researchers or stakeholders wished to be an author on this manuscript or to attend a conference to share the results. While they were all happy for the results to be shared and to have their contribution represented through film and quotes, they strongly felt that the academic sphere was not their domain. They did not feel that being an author on an academic paper was meaningful for them, instead valuing other markers of their contributions, such as a certificate of participation and a copy of the zine as things they could share with others in their network. This suggests additional work is needed, both in making academic spaces more welcoming and accessible for co‐researchers and in developing the confidence of co‐researchers to engage with these new settings.

### 6.1 Strengths and Limitations

This study addresses a significant gap in the inclusive research literature by examining how people with intellectual disabilities can be meaningfully involved in research dissemination. Strengths include involving co‐researchers throughout the dissemination process, the transparent adaptation of the CERD framework and the use of qualitative evaluation to generate practical recommendations for future inclusive dissemination activities.

A number of limitations have also been identified. Throughout the research project, participants were predominantly White British ethnicity and from a narrow range of cultural backgrounds. While people involved in the project represented a broad range of diversity in other areas (such as additional physical and mental health conditions, socio‐economic diversity and a wide age range), we acknowledge that diversity across ethnic and cultural backgrounds was not achieved in this project. While the ethnic and cultural backgrounds of participants largely mirrored the local population from a rural part of England, they limit the transferability of the findings.

Within this study, members of the research team acted as both facilitators of the project and were involved with its evaluation. This has implications for how objective people were able to be both in terms of reporting methodological flaws and limitations of the research and how honest participants were able to be in critiquing the project. Having external researchers conducting the interviews and focus groups would have avoided feelings of loyalty towards the person interviewing participants and may have resulted in more unbiased, critical feedback. Results from participants were largely positive, which suggests that bias or a lack of ability to report issues and difficulties may have been at play due to the preexisting relationship between participants and the interviewer. Additionally, having a researcher (O.H.) act as both interviewer and taking primary responsibility for the analysis of the qualitative interviews presents a dual role which may have influenced interpretation. This dual role arose due to limited resources and having a small research team. While steps were taken to mitigate this issue, it remains a limitation of the study.

The study took place across one site and a single research team. Therefore, the generalisability of the findings cannot be assumed. As described above, an additional limitation is the lack of formal evaluation of the impact of the dissemination materials and a lack of long‐term follow‐up.

This paper suggests that people with intellectual disabilities can and should be involved in the planning, creating and disseminating of research findings. However, detailed guidance on how to best accomplish this is not readily available for people with intellectual disabilities. Therefore, based on our discussions and experiences as a research team, we put forward a number of strategies that may facilitate this process for research teams (see Table 5). While all of these strategies arose from our experiences in this research project, these included wider team discussions and therefore should be viewed as a practice‐informed synthesis generated through the study findings, stakeholder discussions, iterative adaptation of materials and processes and the research team's experiential learning during project delivery. Given the interconnected nature of these influences, recommendations are not attributed to individual data sources.

**TABLE 5:** Recommendations for facilitating including research dissemination with people with intellectual disabilities.

Ideally work with co‐researchers with intellectual disabilities who have been involved throughout your research project. If this is not possible, try to include people who would have been eligible to participate in your study and people from your local community.
If you already have an established co‐research team, it may be helpful to add in a couple of new co‐researchers with intellectual disabilities at the dissemination phase as they will have a fresh perspective on the study. Having to explain your study to new people in accessible language will be helpful when preparing your materials.
Think together as a research team about the wide range of audiences that need to know about your research. Each audience will have a slightly different focus in terms of what they want to know and how they want this to be communicated. You might choose to focus on just one or two of these audiences in your dissemination, which will help this feel more manageable.
Try to make sure dissemination happens early, frequently and throughout your research project. Share results when they become available rather than waiting to the end of a long project. Waiting several months or years for findings can make them seem irrelevant. If necessary, share results informally at first and then again when research articles have been published.
Dissemination is frequently not adequately planned for and costed throughout a research project. Time and energy are scarce at the end of a project as team members move on to other roles. We suggest the having a research team member responsible for dissemination throughout the project, with a clear dissemination plan added to Gantt chart and other project planning materials.
Think with your co‐researchers about how they would like to be involved with dissemination at the beginning. Look at examples of creative ways people have been involved and think about which materials most appeal to them.
Support co‐researchers to plan the dissemination materials they want to create, drawing on their strengths and interests. Get external support to facilitate this if you do not have the necessary research experience or skills.
Using film and animation can be a good way of capturing people's experiences and sharing them widely, especially for those co‐researchers who do not wish to attend conferences or share their experiences in person.

Our project has not yet evaluated the impact of these dissemination materials, to understand how they are received by others, how accessible and engaging they are for each dissemination audience and what impact they have had (if any) on people's willingness to be involved in future research projects or their trust in research or healthcare more broadly. As conceptualised by Langley et al.'s (2018) model of collective making, this study clearly demonstrated the influence of co‐creation on participants and co‐researchers (as reflected in the focus groups and interviews) and on the knowledge discovered and shared and how this is shared (through the range of creative materials produced and the dissemination strategy developed by the research team). However, we have not gone on to evaluate the third domain of influence, that of the mobilisation or activation of this knowledge. This is in line with findings from Chapman et al.'s (2025) scoping review that focused on the lack of evidence that co‐created dissemination leads to changes in health behaviours and outcomes.

Grindell et al. (2022) suggest that the process of co‐creating adaptable, visible and tangible outputs helps participants see that their knowledge and ideas have been heard and valued. This may lead to participants having more trust in the process and achieving reciprocity by producing relevant and acceptable outputs that meet everyone's needs. However, the subsequent impact on health outcomes is yet to be established. It may be that frameworks for inclusive dissemination (such as the CERD) could be extended to consider models of health communication, allowing researchers to address questions such as ‘what do we want to communicate?’ and ‘what do we want people to do differently based on this information?’

Dissemination using inclusive research models is starting to be explored with people with intellectual disabilities and could transform how research is communicated to the client group, with potential implications for creative dissemination materials and subsequent research engagement. Evaluating what impact this may have on health outcomes remains a challenge to be addressed. Our central recommendation is that dissemination should be co‐created with stakeholders from the outset, rather than translating findings into pre‐determined outputs at the end of a project. Creative, flexible and accessible collective‐making approaches can help ensure dissemination materials are meaningful, engaging and usable for diverse audiences.

## Funding

This work was supported by the National Institute for Health and Care Research (NIHR300501).

## Ethics Statement

Full ethical approval for the study was obtained, as detailed within the manuscript.

## Consent

All participants gave written informed consent to take part in focus groups and interviews and for the anonymised use of their quotations to be included in this manuscript.

## Conflicts of Interest

The authors declare no conflicts of interest.

## Supporting information


**Supplementary Material 1.** Co‐MAID infographics.

**Supplementary Material 2.** Easy read summary the co‐MAID research project.

## Data Availability

The data that support the findings of this study are available on request from the corresponding author. The data are not publicly available due to privacy or ethical restrictions.
