# Supplementary material for: Designing and Delivering a Dissemination Programme With Co‐Researchers With Intellectual Disabilities
Source: J Appl Res Intellect Disabil. 2026 Oct 2;39(5):e70313. doi: 10.1111/jar.70313 (PMC13631806; doi:10.1111/jar.70313)
Supplement: Supplementary file 1 [file JAR-39-e70313-s001.docx]

**Supplementary Material 2 – Easy Read Summary**

**The Co-MAID research project**

| **What was this project about?** | |
| --- | --- |
|  | Our project was about making an intervention to help people with anxiety. |
|  |  |
|  | We want to help people change their mental images (pictures in their heads) so they feel less anxious. |
|  |  |
| **What did we do?** | |
|  | First, we did some interviews to see if people with intellectual disabilities can have mental images (pictures in our heads) |
|  |  |
|  | People with intellectual disabilities were good at this! They made clear, detailed mental images (pictures in their heads) |
|  |  |
|  | We found out what helps us to have good mental images. Examples were having instructions we understand and asking us questions to help develop our images. |
|  |  |
|  | We held a big workshop to bring lots of people together. We thought together about what to include in our anxiety intervention. |
|  |  |
|  | Our intervention is called Co-MAID. This stands for a Co-Designed Mental Imagery intervention for People with mild to moderate Intellectual Disabilities. |
|  |  |
|  | We had lots of ideas to make Co-MAID easier for people with intellectual disabilities to understand. |
|  |  |
| **Testing out Co-MAID** | |
|  | Olivia tried out Co-MAID with 6 people in the NHS |
|  |  |
|  | Most people enjoyed the sessions. They felt more relaxed afterwards |
|  |  |
|  | They said mental images were easy to change. |
|  |  |
|  | They liked using apps to practice breathing exercises and record how they feel every day. |
|  |  |
| **What did we learn?** | |
|  | What have we learnt from our research? |
|  |  |
|  | - Doing research is fun! People with learning disabilities enjoyed doing research. |
|  |  |
|  | - We can help some people who feel anxious to feel better by changing their mental images. |
|  |  |
| **What is next?** | |
|  | Now we have money to try out the Co-MAID intervention with 40 people in the NHS. |
|  |  |
|  | People with learning disabilities and other researchers will help Olivia to do this. |
