# Supplementary material for: Designing and Delivering a Dissemination Programme With Co‐Researchers With Intellectual Disabilities
Source: J Appl Res Intellect Disabil. 2026 Oct 2;39(5):e70313. doi: 10.1111/jar.70313 (PMC13631806; doi:10.1111/jar.70313)
Supplement: Supplementary file 2 [file JAR-39-e70313-s002.docx]

Supplementary Materials: Co-MAID Inforgraphic
